# *Larix decidua* Bark as a Source of Phytoconstituents: An LC-MS Study

**DOI:** 10.3390/molecules22111974

**Published:** 2017-11-15

**Authors:** Valeria Baldan, Stefania Sut, Marta Faggian, Elena Dalla Gassa, Sara Ferrari, Gabriele De Nadai, Stefano Francescato, Gianni Baratto, Stefano Dall’Acqua

**Affiliations:** 1 Department of Pharmaceutical and Pharmacological Sciences, University of Padova, Via Marzolo 5, 35131 Padova, Italy; valeria.baldan.4@phd.unipd.it (V.B.); stefania_sut@hotmail.it (S.S.); elena.dallagassa@studenti.unipd.it (E.D.G.); 2 Unifarco spa, Via Cal Longa 62, Santa Giustina 32035 Belluno, Italy; sara.ferrari@unifarco.it (S.F.); gabriele.denadai@unifraco.it (G.D.N.); stefano.francescato@unifraco.it (S.FR.); gianni.baratto@unifraco.it (G.B.); 3 Unired srl, Via N. Tommaseo 69, 35131 Padova, Italy; marta.faggian991@gmail.com

**Keywords:** *Larix decidua* bark, procyanidins, spiro-polyphenol, DPPH, LC-MS, flavonoids, polyphenols

## Abstract

*Larix decidua* bark is a waste of the timber industry and is widely diffused in Northern Italy. This material can be considered a good source of antioxidants and phytoconstituents with possible use in cosmetic or nutraceutical products. In this study, simple extraction of larch bark was performed using mixtures of ethanol/water. Furthermore, the phytochemical composition of larch bark extract was studied using LC-MS^n^ methods and the main constituents were identified as flavonoids, spiro-polyphenols, and procyanidins. To confirm the identification by LC-MS semi-preparative HPLC was performed in order to isolate the main constituents and verify the structures by ^1^H-NMR. Antioxidant properties were studied using an in vitro approach combining DPPH assay and LC-MS in order to establish different roles of the various classes of phytochemicasl of the extract. DPPH activity of some of the isolated compounds was also assessed. The overall results indicate this waste material as a good source of antioxidant compounds, mainly procyanidins, whichresulted the most active constituents in the DPPH assay.

## 1. Introduction

Larch is an industrially important tree, characterized by a strong, water-resistant, and durable wood. Barks and branches are waste material of the timber industry with important, but also partially underexplored, supplies of biologically-active compounds. Several conifer barks contain phytoconstituents, such as tannins, terpenes, and polyphenols [1,2,3]. Many studies have also considered such materials related to their essential oil composition [4]. In the past years our research group has investigated the opportunity to extract phytochemicals from barks and in previous papers the Nepalese *Abies spectabilis* was evaluated for its peculiar content in triterpene derivatives [5] and as a potential source of antioxidants [6].

In this work, we focused on *L. decidua* (European larch), a deciduous conifer endemic to Europe, naturally occurring across central Europe from the Alps, in Eastern France, through the Carpathians, Slovenian mountains, to Southern Poland, Western Ukraine, and Northern Romania. Larch wood is robust, waterproof, and durable, but also flexible in thin strips. The heartwood is particularly weatherproof and, therefore, mainly used as construction timber. Due to its high resin content and difficult machinability, its use for furniture production is limited [1]. Larch sawdust is a byproduct of larch wood production. At present, larch sawdust is mainly used for the production of pellet fuels and contains phenolic compounds, such as lignans and flavonols (mainly dihydroquercetin and dihydrokaempferol) [4].

Arabinogalactans are constituents of larch and these are a class of long, densely-branched polysaccharides with molecular weights ranging from 10,000–120,000 Da. Larch arabinogalactan is considered a good source of dietary fiber, and has been approved as such by the FDA [7]. It also has potential therapeutic benefits as an immune-stimulating agent and as cancer protocol adjunct [7].

Larch has many traditional uses, as an anthelmintic, diuretic, laxative, and vulnerary remedy. Extract of larch bark has been considered to possess diuretic properties, and powdered bark has been used as external application to promote the healing of purulent wounds, and also in chronic eczema and psoriasis [8]. From the tree barks a resinous material rich in terpene can be extracted, and from *L. decidua* Venice turpentine is obtained. In European folk medicine, Venice turpentine is used for preparations for internal and external ailments that are used for renal affection, as an antiseptic, and as an expectorant in chronic bronchitis. It is important to use only small amounts, since even moderate amounts can cause kidney damage, internally, and swelling blisters, externally [8,9].

In the present study, larch bark was used to prepare extracts using solvents with low environmental impact. The obtained extracts were investigated using HPLC-DAD-MS and HPLC-HILIC-MS methods allowing to characterize the polyphenols and procyanidins content. Total phenolic content was determined using the Folin-Ciocalteau reagent method. Isolation of some phenolic constituents as quercetin-3-*O*-glucoside, procyanindins, and major spiro-polyphenols was also performed and the structures were elucidated by NMR and MS techniques. DPPH-HPLC-DAD-MS analysis allowed screening of major antioxidants; spectrophotometric DPPH assay was also used to determine which compounds have higher antioxidant activity and the scavenging capacity of the total extract.

The aim of the study was to evaluate the opportunity to use the larch bark, a waste material of the wood industry, as a source of valuable compounds with an eco-friendly approach; to obtain it we prepared extracts using only ethanol as the organic solvent and water.

## 2. Results and Discussion

The extraction of bioactive compounds from plant materials is the first step in the utilization of phytochemicals in the preparation of dietary supplements or nutraceuticals, food ingredients, pharmaceutical, and cosmetic products [10]. Extracts from the bark of different conifer species are known to contain various polyphenols, tannins, and procyanidins [11]. The selection of extracting conditions and solvents affect the extract composition; different alcohol-water mixtures have been tested to select the efficient solvent for flavonoids and procyanidins extraction [10]. We considered aqueous-ethanol mixtures at 0°, 40°, 60°, and 80°, extracting under stirring for 30 min at room temperature.

The polyphenol content of the dried bark extracts was initially analyzed by HPLC-DAD and the classes of phenolics detected were determined on the basis of the UV spectra of each peak compared with reference compounds, namely, rutin, chlorogenic acid, gallic acid, and catechin. This preliminary investigation led to the observation of the presence of flavonoids and small amounts of catechins, while chlorogenic acid and gallic acid derivatives were not detectable. Quantitative estimation of flavonoid and catechin contents are reported in Table 1.

Procyanidins (PACs) were analyzed using HPLC-HILIC method [6] using a fluorimeter and mass spectrometer; the separation allowed distinguishing different procyanidin groups, eluting at higher retention times corresponding to the degree of polymerization. On the basis of the MS data the chromatogram was divided into four parts corresponding to polymeric classes of PACs, as indicated in Table 2. The total PACs amount varies from 5.66% to 7.59% with a heterogeneous class composition and a higher amount of dimers (Table 2); quantification was obtained using PAC-B2 as the reference standard.

The larch bark contains small amount of polyphenol compounds with 0.02% of flavonoids. Bark extracts instead show higher flavonoid content, with a total amount that varies from 0.51% to 0.80%; extraction procedure results are necessary to increase the phytochemicals final content, allowing to increase up to seven times the total amount of polyphenols.

The different ethanol-water mixtures allowed an extraction yield ranging from 1.4% of weight for aqueous extract and 6.4% for ethanol 80°; 40° and 60° ethanol extracts show a similar yield of extraction and analogous content of flavonoids. The most efficient mixture for the extraction of flavonoids was 40°, and for procyanidins, was 80° ethanol extract. 

The total phenolic and total flavonoids contents were also measured using a Folin-Ciocalteau (FC) assay. Phenolic compounds have redox properties, which allow them to act as antioxidants [12]. As their free radical scavenging ability is facilitated by their hydroxyl groups, the total phenolic concentration could be used as a basis for rapid screening of antioxidant activity. Flavonoids, including flavones, flavanols, and condensed tannins (such procyanidins), are plant secondary metabolites, the antioxidant activity of which depends on the presence of free OH groups, especially 3-OH. Results are expressed as the percentage of rutin, used as a reference compound (Table 3).

The obtained data revealed differences in total reducing capacity of the extracts. The FC reagent measures a sample’s reducing capacity, but this is not only reflected in the total phenolic profiles [13]. This method suffers from a number of interfering substances present in plants, such as ascorbic acid, sulfur-containing compounds, mono- and disaccharides, etc. Despite its limitations for quantifying phenolic compounds in plant extracts, the FC method is the recommended method for the measurement of total reducing capacity [14].

In view of an eco-friendly extraction, and considering both the weight yield and the amount of extracted compounds, we decided to proceed with the characterization and antioxidant activities only using the 40° ethanol extract that has the lowest use of organic solvent. 

In the proceeding of the study we performed the identification of constituents with HPLC-MS^n^ analysis based mainly on fragmentation patterns by comparison of reference compounds, or with literature data [15]. Identified compounds are reported in Table 4. 

In order to establish and confirm the structure of constituents in the larch extract, fragmentation pathways were studied using the LC-MS^n^ approach; the total extract was also subjected to semi-preparative HPLC-UV analysis in order to identify the structure of constituents in larch bark that lack literature data for MS/MS measurements. With the method described below, we obtained isolated compounds that were subsequently characterized by ^1^H-NMR using assignments of previous studies [16,17,18].

Related to compounds that are not identified on the basis of literature data, comprehensive MS/MS analysis and NMR of isolated compounds were performed.

The MS results show the presence of three peaks presenting a pseudo-molecular ion at *m*/*z* 541 with a similar fragmentation pathway. To establish the possible structure of these compounds, small amounts were isolated (1–5 mg) by semi-preparative HPLC; the ^1^H-NMR were then acquired on dried residue of the isolated products. Compound eluting at 12.36 min shows the diagnostic resonances at δ 4.58 (H-2) and δ 4.07 (H-3), as well as the singlet at δ 6.08 (H-6), thus indicating that its structure can be assigned to larixinol (2*R*,2′*R*,3*R*,3′*R*)-3′,4,5′,6-tetrahydroxy-2,2′-bis(4-hydroxyphenyl)-3′,4′-dihydro-2′*H*-spiro(1-benzofuran-3,9′-furo(2,3-*h*)chromen)-8′-one [18]. The isomeric compound that eluted at 11.16 min presents the same behavior in MS, but at the ^1^H-NMR signals at δ 4.60 (H-2) and 4.22 (H-3) being ascribable on the basis of literature data to 3,2′-*epi*-larixinol, while the third peak presented the same *m/z* values and ^1^H-NMR signals that support the presence of 3-epilarixinol. The ^1^H-NMR data of the compound were in good agreement with the literature [18].

**Figure 1:**
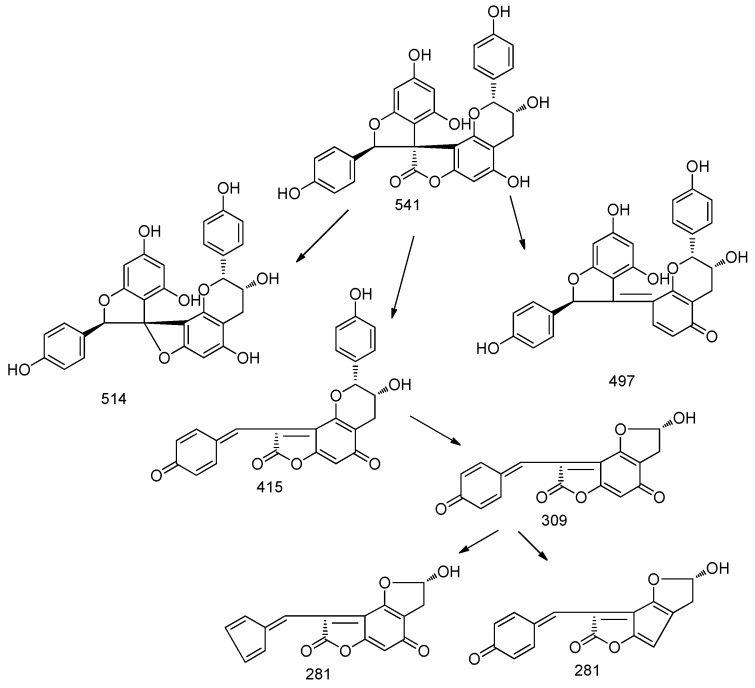

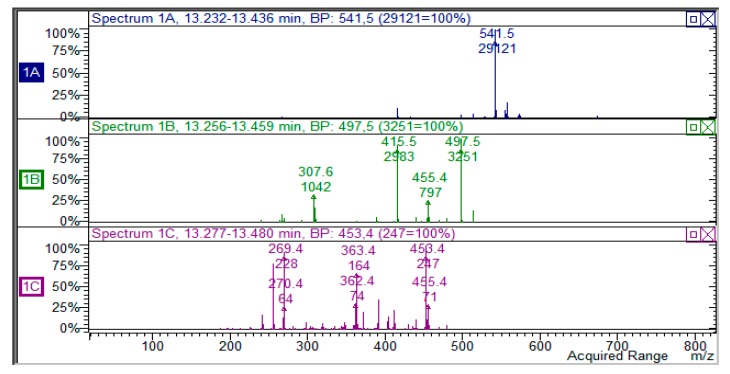
The proposed fragmentation pattern of larixinol.

Thus, we studied the main fragmentation pathway for these derivatives in ESI negative mode. The pseudomolecular ion at *m*/*z* 541 in MS^2^ is characterized by the loss of 27 and 44 Da; further significant fragments revealed the loss of 126 Da and formation of quinone derivatives, at *m*/*z* 415 and 309 that further showed a loss of 28 (CO), yielding the fragment at *m*/*z* 281 (Figure 1). 

**Figure 2:**
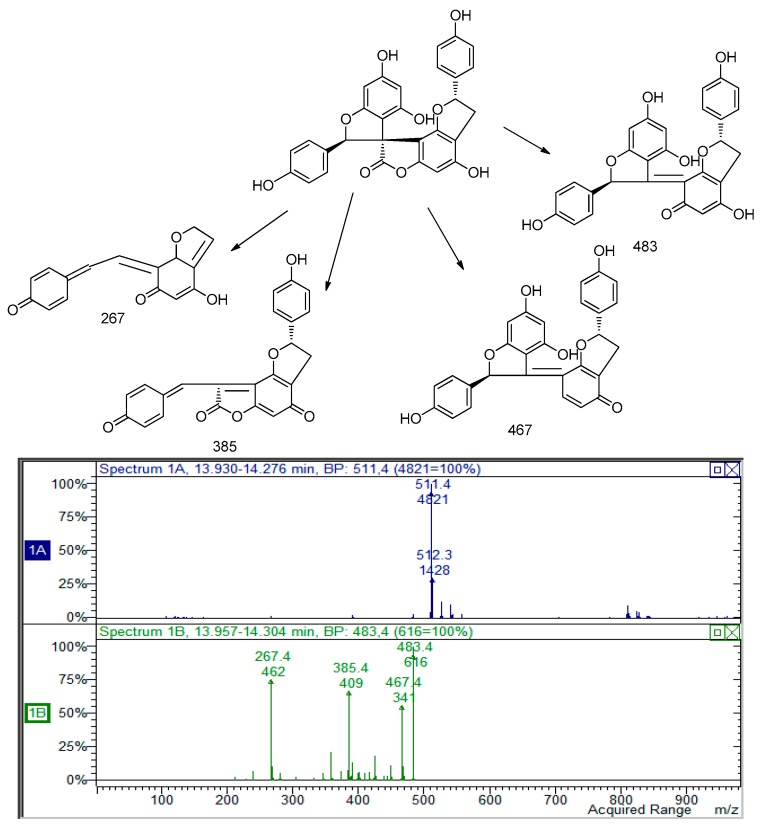
The proposed fragmentation pattern for the derivative that was tentative assigned to 4,4′,6′-trihydroxy-2,2′-bis(4-hydroxyphenyl)-2*H*,2′*H*-spiro(benzo(1,2-*b*:3,4-*b*)difuran-8,3′-benzofuran)-7(3*H*)-one.

Similar behavior in MS/MS fragmentation pathways were observed for the peak at 13.29 min presenting *m*/*z* 511. In particular, the loss of 27 and 44 Da and the retro diels-alder reaction support the presence of a structure similar to larixinol. The loss of 30 Da suggests the lack of a carbon atom and a hydroxyl group and we suggest the presence of a second unit of 2-(4-hydroxyphenyl)-2,3-dihydrobenzofuran-4-ol moiety. The structure of this compound was tentatively assigned to 4,4′,6′-trihydroxy-2,2′-bis(4-hydroxyphenyl)-2*H*,2′*H*-spiro(benzo(1,2-*b*:3,4-*b*′)difuran-8,3′-benzofuran)-7(3*H*)-one; the MS and MS^2^ spectra are indicated in Figure 2.

**Figure 3:**
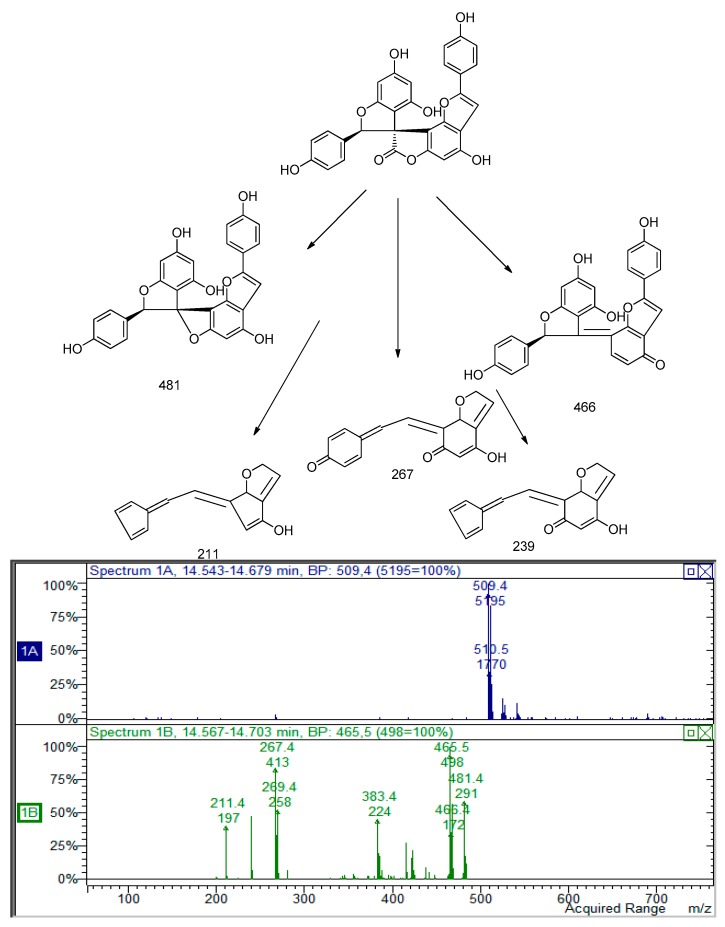
The proposed fragmentation pattern for the derivative (2′*R*,3′*R*)-4,4′,6′-trihydroxy-2,2′-bis(4-hydroxyphenyl)-2′*H*,7*H*-spiro(benzo(1,2-*b*:3,4-*b*′)difuran-8,3′-benzofuran)-7-one.

The derivative with *m*/*z* 509 also presented a similar fragmentation pathway. The loss of 27 and 43 Da were observed, as in the previous derivative, and as in the larixinol. Furthermore, the loss of 126 Da corresponding to the 1,3,4 trihydroxy benzene and the similar rearrangement indicated for the other spiro-polyphenols, suggest the structure of the compound as a dehydro derivative of the previous one. The structure of this compound was proposed as (2′*R*,3′*R*)-4,4′,6′-trihydroxy-2,2′-bis(4-hydroxyphenyl)-2′*H*,7*H*-spiro(benzo(1,2-*b*:3,4-*b*′)difuran-8,3′-benzofuran)-7-one. The MS and MS^2^ spectra and the possible fragmentation pathways are reported in Figure 3.

**Figure 4:**
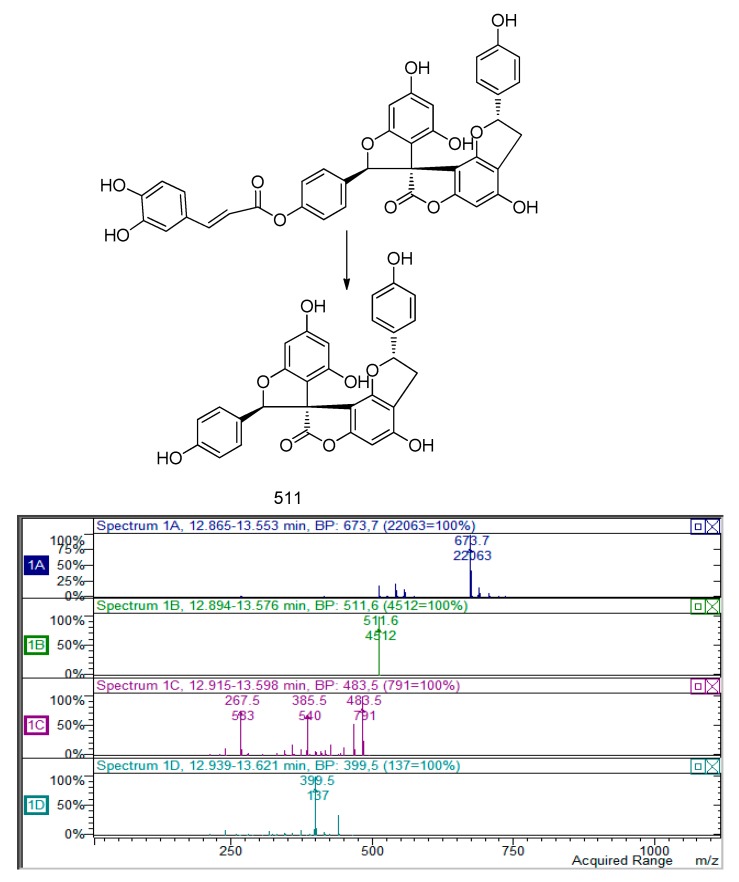
Proposed fragmentation pattern of the unknown compound with *m/z* 673.

Other peaks at 10.63 and 11.50 min presenting an *m*/*z* value of 673 was assigned to a putative derivative of previous compounds. The MS^2^ presents a fragment ion at *m*/*z* 511 and a further fragmentation pathway that resume previous to the one compound (Figure 4).

**Figure 5:**
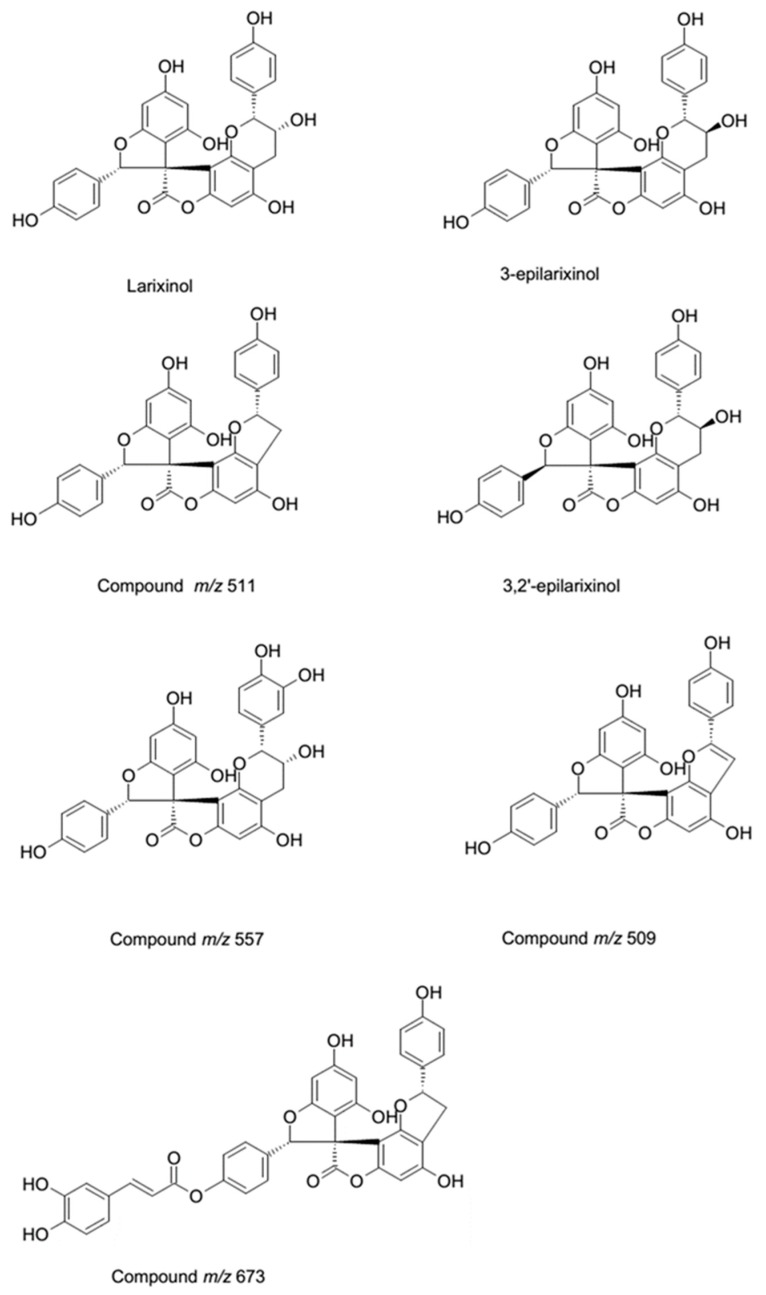
Proposed structures of spiro-polyphenols in *L.decidua* bark extract.

The amount of derivatives isolated up to now from compounds at *m*/*z* 511, 509 and 673 were not sufficient to record by H-NMR. Isolation of these derivatives is in progress. Structures of spyro-polyphenols are reported in Figure 5.

DPPH was used to evaluate the different antioxidant activity of compounds in the bark extract. Different aliquots of extract were incubated for one hour with an increasing amount of a DPPH solution (3 mg/100 mL). The extract: DPPH ratio solutions was 1:2, 1:3, 1:10. The samples were then analyzed in HPLC-DAD-MS^n^ comparing peaks with the untreated extract; in this way we obtained a qualitative view of compounds reacting with DPPH. The increasing of the content of DPPH, and the keeping of the extract amount constant, allowing to obtain chromatograms in which the peaks are not completely deleted; this procedure allows to identify which compounds react earlier and, thus, a scale of free scavenging activity.

Peak areas (PAs) of the untreated sample were set as 100% and the relative PAs of the sample that were reacted with DPPH of different concentrations were calculated; the decrease in PAs was determined. Table 4 reports the identification of constituents of 40° ethanol larch bark extract, the amount of each compound (% *w*/*w*) calculated from HPLC-DAD data, and the results of the DPPH assay expressed as the percentage of the PA decrement compared to the reference sample for the three ratios considered (1:2; 1:3; 1:10).

It is believed that the reaction between an antioxidant and a free radical resulted in the oxidation of the antioxidant. In the case of polyphenols, the transfer of one or more hydrogen atoms from the antioxidant to the free radical was involved [8]. Once reacted, the conjugated system in the molecular structure would be destroyed. On this basis, after the interaction with DPPH, the PAs of the free radical scavenging compounds would decrease in the HPLC-DAD chromatogram while, for those without antioxidant effects, there was almost no change in their PAs after spiking with DPPH solution [19].

Procyanidins and some larixinol derivatives showed a sharp decrease of PAs, with a decrement up to 21.50% for procyanidin tetramer B-type even at low amounts of DPPH solution. At the increase of the DPPH solution, a linear trend of a decrease with values that reach up to almost 60% can be observed. Flavonoids, instead, are not so involved in the antioxidant capacity, with a PA decrement of 18% at most.

**Figure 6:**
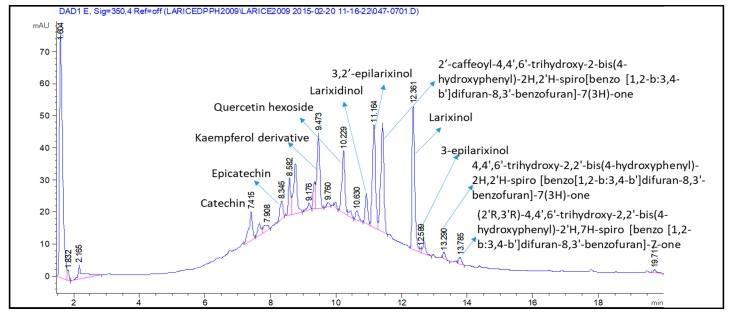
HPLC-DAD chromatogram with identified compounds.

**Figure 7:**
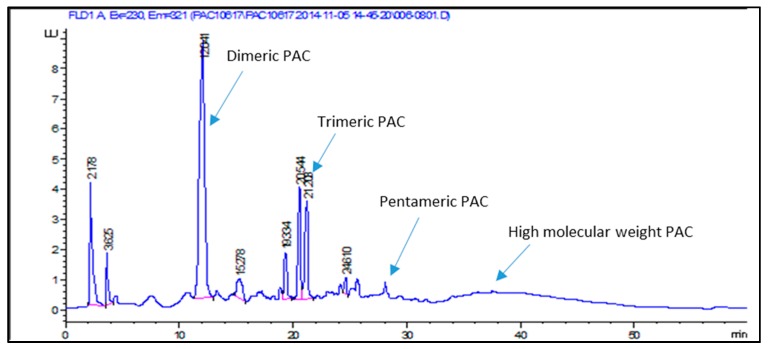
HPLC-HILIC-FLD-MS of identified proanthocyanidins in *L.decidua* bark extract.

Some constituents, like catechin, isorhamnetin, and other compounds in low quantity were not taken into account because it was not possible to follow their decrement. Exemplificative chromatograms are reported in Figure 6 and Figure 7.

The antioxidant properties of some of the isolated compounds were also evaluated using a spectrophotometric DPPH assay. Quercetin glucoside, ascorbic acid, and PAC B2 were used as reference compounds (Table 5).

The obtained results show that total bark extract presents a scavenging capacity comparable to ascorbic acid, used as a reference substance. Observing the IC_50_ values of the two isolated compounds, it can be noted that these are not so involved in the antioxidant capacity shown by the total extract; in fact, larixinol and its derivative show higher IC_50_ values. Related to the isolated compounds, high activity was observed for quercetin derivative and procyanidin B2; such compounds present the catechol group that is known to strongly increase the activity in the DPPH assay [14]. Considering the measured IC_50_ values, and considering the amount in the extracts, we can conclude that the main antioxidant activity of larch bark extract observed in the DPPH assay can be ascribed to procyanidins that are present in higher amount than other polyphenols. This is also confirmed by the data obtained with DPPH-HPLC-MS analysis, in which procyanidins show a rapid reaction towards DPPH solution even at low concentration. 

## 3. Materials and Methods

### 3.1. Plant Material

Larch bark was kindly provided by Unifarco spa, Belluno, Italia. A voucher specimen (LB012017) is deposited at Department of Pharmaceutical and Pharmacological sciences of the University of Padua.

### 3.2. General Materials

Solvents used for analysis and for extracts preparation, DPPH and Folin-Ciocalteau reagent were purchased from Sigma Aldrich (St. Louis, MO, USA); standard references were products of Sigma Aldrich(St. Louis, MO, USA) and Phytolab (Vestenbergsgreuth, Germany).

### 3.3. Extraction and Sample Preparation

Finely-powdered bark was extracted using different water: ethanol mixtures (0°, 40°, 60°, 80° ethanol) under stirring for 30 min at room temperature and with a ratio of drug: solvent of 1:10. The extracts were then filtered and solvents removed to dryness using a rotary evaporator. Dried residues were dissolved in water: ethanol mixtures with a final concentration of 4 mg/mL. Bark samples were prepared extracting in water:ethanol 50:50 mixtures, with a final concentration of 20 mg/mL

### 3.4. High-Performance Liquid Chromatography-Diode Array Detector-Mass Spectrometry (HPLC-DAD-MS^n^)

Quali-quantitative analysis of phenolic derivatives was obtained by HPLC-DAD-MS^n^.

The measurements were performed with an Agilent 1260 chromatograph (Santa Clara, CA, USA) equipped with 1260 diode array (DAD) and Agilent/Varian MS-500 ion trap (Santa Clara, CA, USA) as detectors. Separation was achieved using an Agilent Eclipse XDB C-18 (3.0 × 150 mm) 3.5 μm as stationary phase. The mobile phases were water 0.1% formic acid (A) and acetonitrile (B). The elution gradient started at 90% A, then decreased to 0% over 25 min. The flow rate was 400 uL/min. At the end of the column a T connector split the flow rate to DAD and MS. The DAD detector was used to quantify flavonoids and rutin, chlorogenic acid, and gallic acid were used as reference compounds. The chromatograms were monitored at 280, 330 and 350 nm and the UV-VIS spectra were acquired in the range of 200–650 nm. The sample injection volume was 10 μL. MS spectra were recorded in negative mode in 50–2000 Da range, using an ESI ion source. Fragmentation of the main ionic species were obtained by the turbo data depending scanning (TDDS) function. Identification of compounds was obtained based on the fragmentation spectra, as well as the comparison of the fragmentation pattern with the literature and injection of reference compounds when available. Quantification of phenolic constituents was obtained with the method of the calibration curve: rutin, chloroegnic acid, gallic acid, and catechin (Sigma Aldrich, St. Louis, MO, USA) were used as external standard for polyphenol quantification. Calibration curves were determined using a series of standard solutions in a range of concentrations of 8–80 μg/mL, 10–103 μg/mL, 10–100 μg/mL, and 9–99 μg/mL, respectively; linear regression were as follows: for rutin, *y* = 16.959*x* + 7.7846 (R^2^ = 0.999); for chlorogenic acid, *y* = 26.97*x* − 1.4129 (R^2^ = 0.999); for gallic acid, *y* = 31.47*x* − 110.01 (R^2^ = 0.997); and for catechin, *y* = 5.9718*x* − 2.4041 (R^2^ = 0.999). LOD = 2 μg/mL; LOQ = 6 μg/mL.

### 3.5. High-Performance Liquid Chromatography-Fluorimeter-Mass Spectrometry (HPLC-FLD-MS)

Quantitative analysis of proanthocyanidins was performed with HPLC-FLD-MS on the same system described above but using a fluorimetric detector (1260 series) and MS 500. The fluorescence excitation wavelength was set at 231 nm, and the emission wavelength was 320 nm. As in the case of DAD-MS detectors, a T-connection was fitted out of a chromatographic column and the effluent was split to the fluorimeter and ion trap. Analyses were performed on tosoh TSKgel Amide 80 2.1 mm ID × 15 cm L μ 3.0 as the stationary phase and acetonitrile 0.5% formic acid (A), H_2_O 0.5% formic acid (B) as the mobile phase. A gradient program was used as follows: (0 → 20th min: A:B (99:1) → A:B (80:20) 20 → 25th min: A:B (80:20) → A:B (80:20) 25 → 45th min: A:B (80:20) → A:B (35:65) 45 → 51th min: A:B (35:65) → A:B (20:80) 51 → 52th min: A:B (20:80) → A:B (99:1) 52 → 60th min: A:B (99:1) → A:B (99:1)). The flow rate was 200 μL/min. Quantitative analysis was carried out using a calibration curve of procyanidin B2; the calibration curve was obtained in a concentration range of 2–50 μg/mL with a linear regression as follows: *y* = 15.006*x* + 9.6184 (R^2^ = 0.9998). LOD = 0.5 μg/mL; LOQ = 5 μg/mL.

### 3.6. Preparative Liquid Chromatography

The LC preparative system consisted of a Varian 920 chromatograph with a quaternary pump; chromatographic separation was performed on an Agilent C18 column (2.1 × 250 mm, 5 micron). The mobile phase was delivered at a flow rate of 2.5 mL/min. The chromatographic run was performed with a binary, linear A/B gradient (solvent A: aqueous formic acid 0.1%; solvent B: acetonitrile). The program was as follows: 0 min 75% B; 22 min 35% B; 22.5 min 35% B; 23 min 75% B and isocratic up to 26 min. The injection volume was 200 μL. Each peak was collected and the obtained fractions were evaporated to dryness under vacuum in a rotary evaporator at 60 °C. The dry residues were re-dissolved in deuterated methanol for ^1^H-NMR analysis.

NMR spectra were obtained in a Bruker Avance III spectrometer, using standard pulse sequences dissolving samples in deuterated methanol.

The isolated compounds are larixinol, 4,4′,6′-trihydroxy-2,2′-bis(4-hydroxyphenyl)-2*H*,2′*H*-spiro(benzo(1,2-*b*:3,4-*b*′)difuran-8,3′-benzofuran)-7(3*H*)-one, (2′*R*,3′*R*)-4,4′,6′-trihydroxy-2,2′-bis(4-hydroxyphenyl)-2′*H*,7*H*-spiro(benzo(1,2-*b*:3,4-*b*′)difuran-8,3′-benzofuran)-7-one, and an unknown compound with *m*/*z* 673 [16,17,18].

### 3.7. Folin-Ciocalteau Assay

Total phenolic amount in different extracts was determined by spectrophotometric method using Folin-Ciocalteau reagent. Protocols were previously used by our group [14]. Briefly, 5 mL of distilled water, 0.5–1.0 mL of sample, and 1.0 mL of Folin-Ciocalteau reagent was added to a 25 mL flask. Next, 10 mL of sodium carbonate saturated solution was added, followed by distilled water. Solution mixture was allowed to stand in the dark at room temperature for 15 min; the absorbance was recorded at 725 nm spectrometrically. Total phenolic content was standardized against rutin, expressed as percentage *w*/*w*.

### 3.8. DPPH Assay

The free radical scavenging ability of extracts against DPPH (1,1-diphenyl-2 picrylhydrazyl) free radicals were evaluated. The methanolic DPPH solution (50 μg/mL) was prepared daily. Then, 1 mL of 2-, 5-, or 10-fold diluted extract in methanol was mixed with 3 mL of DPPH solution and incubated for 30 min in the dark and the absorbance was taken at 517 nm. A standard curve was prepared by determining the decrease in absorbance of the DPPH radical solution. The results were expressed as IC_50_ (μg/mL).

DPPH solution was also used to evaluate the scavenging properties of the main polyphenolic compounds in HPLC-MS using the method previously described [14,20] with minor modifications. Five-hundred microliters of total extract was incubated with increasing volume of DPPH solution in ratios of 1:2, 1:3, and 1:10. The samples were incubated for one hour and were then analyzed by HPLC-DAD-MS and HPLC-HILIC-MS, with the same system and method described above, to determine which peaks have higher antioxidant activity.

## 4. Conclusions

The analysis of polyphenolic compounds in hydroalcoholic bark extract of larch shows a prevalence of flavonoids and B-type proanthocyanidins with total amounts of 0.8% and 6.31%, respectively. The subsequent MS characterization allowed identifying the main flavonoidic compounds as spiro-polyphenols. Larixinol is the main spiro-biflavonoid and it is present in different epimeric forms and derivatives. In the present study, we tentatively assigned a fragmentation pattern for some of these compounds. Isolation and identification is ongoing for larixinol derivatives not yet identified.

We have also evaluated the scavenging capacity towards DPPH using a HPLC-DAD-MS method with the identification of the specific compounds that have scavenging capacity. The PAs of procyanidin and larixinol were significantly reduced, indicating that these possess antioxidant effects; considering the results obtained with the spectrophotometric DPPH assay, procyanidins result in being the main substance responsible for antioxidant activity of larch bark extract. Larch bark extract shows an antioxidant activity comparable to ascorbic acid, which is known to be a powerful antioxidant. In depth studies are needed, but the overall results indicate that the bark of the larch is a good source of antioxidant compounds.

## Figures and Tables

**Table 1:** The amount of flavonoids and catechins in larch bark and larch bark extracts obtained with different degrees of aqueous ethanol, and extraction yield.

	% *w*/*w*		
Sample	Flavonoids	Catehin and Epicatechin	Yield of Extraction
Larch Bark	0.02 ± 0.01		-
40° Extract	0.80 ± 0.02	0.06 ± 0.02	5.61 ± 0.01
60° Extract	0.73 ± 0.01	0.08 ± 0.02	5.95 ± 0.01
80° Extract	0.57 ± 0.01	0.05 ± 0.01	6.40 ± 0.01
Aqueous Extract	0.51 ± 0.01	0.03 ± 0.01	1.42 ± 0.01

**Table 2:** The amount of different classes of procyanidins in larch bark and larch bark extracts obtained with different degrees of aqueous ethanol.

% *w*/*w*					
Sample	Monomers	Dimers	Trimers/Tetramers	Pentamers/Hexamers	Total
Larch bark	0.18 ± 0.02	0.52 ± 0.01	0.15 ± 0.01	0.32 ± 0.03	1.17
40° Extract	2.04 ± 0.19	1.77 ± 0.14	1.03 ± 0.05	1.47 ± 0.15	6.31
60° Extract	2.54 ± 0.16	2.26 ± 0.10	1.22 ± 0.04	1.53 ± 0.01	7.59
80° Extract	3.12 ± 0.12	1.88 ± 0.06	1.22 ± 0.08	1.69 ± 008	7.61
Aqueous Extract	2.19 ± 0.09	1.36 ± 0.06	0.97 ± 0.01	1.15 ± 0.03	5.66

**Table 3:** Total reducing capacity obtained with the Folin-Ciocalteau method expressed as the percentage *w*/*w* of rutin.

Sample	Total Reducing Capacity (as Rutin %)
40° Extract	20.19 ± 1.59
60° Extract	34.28 ± 0.37
80° Extract	29.85 ± 0.30
Aqueous Extract	16.47 ± 0.52

**Table 4:** HPLC-MS identified compounds in *L. decidua* bark extract (40° ethanol), percentage amount of identified compounds (% *w*/*w*) and decrease of peak areas (PAs) after DPPH reaction, in different ratios (1:2; 1:3; 1:10).

Identification	Retention Time (min)	λmax (nm)	[M − H]^−^	Fragments	% *w*/*w*	% of PAs Decrement (1:2)	% of PAs Decrement (1:3)	% of PAs Decrement (1:10)
Quinic acid	2.85	-	191.3	111	0.013	n.s.	n.s.	n.s.
Procyanidin B2	3.73 **	280	577	451–425	3.00	10.39	35.20	52.61
Procyanidin trimer B	3.80 **	280	865	695–577	2.37	16.00	33.48	55.59
Procyanidin tetramer B	3.95 **	280	1153	1027–577	0.94	21.50	34.13	64.77
Catechin *	7.41	280	289	245–205–179	0.041	n.s.	n.s.	n.s.
Epicatechin *	8.58	280	289	245–205–179	0.022	n.s.	n.s.	n.s.
Kaempferol Derivative	9.47	370	573.6	529–447	0.070	10.46	12.81	17.05
Quercetin-hexoside	10.22	330	447.5	301–179–151	0.084	1.04	5.59	9.78
2′-caffeoyl-4,4′,6′-trihydroxy-2-bis(4-hydroxyphenyl)-2*H*,2′*H*-spiro[benzo[1,2-*b*:3,4-*b*′]difuran-8,3′-benzofuran]-7(3*H*)-one	10.63	328	673.6	511–483–467–385	0.072	13.95	18.62	18.67
Larixidinol	11.01	368	557.6	513–431–269	0.029	n.s.	n.s.	n.s.
3,2′-epilarixinol	11.16	332	541.6	497–415–309	0.119	2.42	5.17	24.49
2′-caffeoyl-4,4′,6′-trihydroxy-2-bis(4-hydroxyphenyl)-2*H*,2′*H*-spiro[benzo[1,2-*b*:3,4-*b*′]difuran-8,3′-benzofuran]-7(3*H*)-one isomer	11.50	336	673.8	511–483–399	0.131	6.04	11.62	49.45
Larixinol	12.36	366	541.6	497–415–309	0.143	8.13	15.82	59.90
3-epilarixinol	12.60	324	541.6	497–415–309	0.015	3.48	8.73	n.s
4,4′,6′-trihydroxy-2,2′-bis(4-hydroxyphenyl)-2*H*,2′*H*-spiro[benzo[1,2-*b*:3,4-*b*′]difuran-8,3′-benzofuran]-7(3*H*)-one	13.29	324	511.5	483–467–385	0.013	054	1.08	2.48
(2′*R*,3′*R*)-4,4′,6′-trihydroxy-2,2′-bis(4-hydroxyphenyl)-2′*H*,7*H*-spiro[benzo[1,2-*b*:3,4-*b*′]difuran-8,3′-benzofuran]-7-one	13.78		509	481–466–267	0.006	n.s.	n.s.	n.s.
Larixinol derivative	14.94		569	541–497–415–309	0.006	1.38	3.45	n.s.
Luteolin *	15.24	348	285.4	269–241	0.003	n.s	n.s	n.s
Isorhamnetin	15.47	345	315.4	300–269–151	0.034	n.s.	n.s.	n.s.

* Catechin and procyanidin dimer B-type were used as reference compounds for the comparison of fragmentation pathway; ** Retention times for proanthocyanindins are referred to HPLC-HILIC-MS chromatogram.

**Table 5:** IC_50_ (μg/mL) values of scavenging capacity.

Sample	IC_50_ (μg/mL)
Total extract 40°	3.93 ± 0.38
Larixinol derivative (*m*/*z* 673)	33.04 ± 0.41
Larixinol	30.54 ± 0.29
Quercetin-glucoside	2.90 ± 0.09
Procyanidin B2	1.38 ± 0.09
Ascorbic acid	3.50 ± 0.02
